# Multi-Hierarchical Fusion to Capture the Latent Invariance for Calibration-Free Brain-Computer Interfaces

**DOI:** 10.3389/fnins.2022.824471

**Published:** 2022-04-25

**Authors:** Jun Yang, Lintao Liu, Huijuan Yu, Zhengmin Ma, Tao Shen

**Affiliations:** School of Information Engineering and Automation, Kunming University of Science and Technology, Kunming, China

**Keywords:** brain-computer interfaces, motor imagery, deep learning, convolutional neural network, bidirectional long short-term memory

## Abstract

Brain-computer interfaces (BCI) based motor imagery (MI) has become a research hotspot for establishing a flexible communication channel for patients with apoplexy or degenerative pathologies. Accurate decoding of motor imagery electroencephalography (MI-EEG) signals, while essential for effective BCI systems, is still challenging due to the significant noise inherent in the EEG signals and the lack of informative correlation between the signals and brain activities. The application of deep learning for EEG feature representation has been rarely investigated, nevertheless bringing improvements to the performance of motor imagery classification. This paper proposes a deep learning decoding method based on multi-hierarchical representation fusion (MHRF) on MI-EEG. It consists of a concurrent framework constructed of bidirectional LSTM (Bi-LSTM) and convolutional neural network (CNN) to fully capture the contextual correlations of MI-EEG and the spectral feature. Also, the stacked sparse autoencoder (SSAE) is employed to concentrate these two domain features into a high-level representation for cross-session and subject training guidance. The experimental analysis demonstrated the efficacy and practicality of the proposed approach using a public dataset from BCI competition IV and a private one collected by our MI task. The proposed approach can serve as a robust and competitive method to improve inter-session and inter-subject transferability, adding anticipation and prospective thoughts to the practical implementation of a calibration-free BCI system.

## Introduction

Brain-computer interfaces (BCIs) (Chiarelli et al., 2018; Emami and Chau, 2018; Zhang et al., 2019) play an essential role as a communication pathway between the human brain and the external world in the situation where the peripheral pathway nerve is severely damaged by diseases such as apoplexy or degenerative pathologies. Owing to progress in neuroscience and computer science in the past decades, BCI has harvested significant developments. Thereby, it has been regarded as a top interdisciplinary research domain in computational neuroscience and intelligence (Gu et al., 2020). Monitoring and decoding information in electroencephalography (EEG) signals and converting it into computer commands are the key tasks of BCI systems. Among the different BCI paradigms, motor imagery electroencephalography (MI-EEG) (Tang et al., 2016; Li et al., 2017) has been considered the most flexible method due to its promising potential in discerning different brain activities. The process of motor imagery (MI) (Kappes and Morewedge, 2016) cued by external vision could trigger the mental simulation, which would involve the event-related desynchronization (ERD) and event-related synchronization (ERS) simultaneously in certain rhythms (Tariq et al., 2017) (μ bands 8–13 Hz and β bands 17–30 hz) of EEG signals at different areas of the cortex. Various brain activities can be used to experimentally detect such a phenomenon (Liu et al., 2018). Electroencephalography is a typical brain activity measuring method with high time resolution.

Consequently, accurate interpretation of EEG signals from the user is a key factor of an MI-based BCI system. Despite the achievement obtained in MI-EEG-based BCI applications, some bottlenecks still hinder its effectiveness and general applicability. First, EEG is a non-stationary signal (Gramfort et al., 2013; Cole and Voytek, 2018) with an exceptionally low signal-to-noise ratio (Repovs, 2010), preventing accurate interpretation of EEG signals. Second, due its characteristics and being different from image data, EEG brings deep learning models’ poor performance to capture appropriate discriminative features from different tasks, especially in multiclass tasks (exceed 2 class). Accordingly, most of the previous works only focused on binary classification, which impeded the control performance of BCI systems. Third, high inter-session and inter-subject variability (Clerc et al., 2016) arise in physiological differences between different periods and individuals. Inevitably, a time-consuming calibration procedure for the BCI system was required. It also limited the popularization of EEG-based BCI.

To address the challenges mentioned above, we propose a novel multi-hierarchical representation fusion (MHRF) framework. It can be used as a supplement with perceptive insights into the relationship between the MI-EEG data and human intention. A joint deep recurrent neural network (RNN) is adopted to learn high-level representation from sequential EEG signals while a CNN is used for learning its spectral image transformation by short-time Fourier transform (STFT). The features generated by bidirectional LSTM (Bi-LSTM) and CNN are fused with the stacked sparse autoencoder (SSAE) to obtain discriminative features. The main contributions of this article can be summarized as follows:

This paper proposes a deep learning decoding method based on MHRF on MI-EEG. It consists of a concurrent framework constructed of bidirectional LSTM (Bi-LSTM) and CNN to fully capture the contextual correlations of MI-EEG and the spectral feature. Also, the SSAE is employed to concentrate these two domain features into a high-level representation for cross-session and subject training guidance. Experimental analysis verifies the validity and practicability of the proposed method by using public data sets from BCI competitions and private data sets collected by MI tasks.

## Related Works

As a specific machine learning (ML) algorithm, deep learning can provide an end-to-end architecture and automatic feature extraction ability. A deep learning model learns general features by lower layers and specific features by higher layers from relevant subjects or sessions. The learned features enable the BCI to process raw data with competitive performance.

Huang et al. (2020) proposed a classification method of EEG signals based on multi-scale CNN model, which used short-time Fourier transform (STFT) to input time-frequency characteristics of EEG data into a multi-scale CNN model for EEG classification. Chen et al. (2021) propose an IS-CBam-CNN, which adaptively extracts the time and frequency distribution information of MI-EEG signals by introducing an attention module to improve the robustness of the decoding model. Lashgari et al. (2021) proposed an end-to-end neural network based on the attentional mechanism and combined with different data enhancement techniques to overcome the problems of low classification accuracy and low data volume in MI-EEG decoding. Li et al. proposed a multi-dimensional MI-EEG decoding method based on time-frequency analysis and Clough-Tocher (CT) interpolation algorithm (Li et al., 2021). Dai et al. (2020) proposed the classification of HS-CNN for MI-EEG. The convolutional kernels of the network have different scales, which can solve the sensitivity of different subjects to the scale of the convolutional kernels. Zhang et al. (2021) proposed an EEG-inception architecture based on CNN for mi-EEG decoding, which uses raw EEG as input and has high accuracy for time series classification.

However, one part of these studies was focused on binary classification tasks, while others were mainly working on binary classification and making an exploratory study for multi-classification; either insufficiency of the point at the fusion and utilization of different domain features or inadequate attention has been paid to individual differences. The fundamental assumption under the ML methods is that training data can cover the probability distribution of the feature space used in the testing applications. However, the assumption is often violated in bioelectric signal processing fields due to obvious variation in EEG induced by differences in physiological structure and psychological states. To compensate for such inter-session and inter-subject variabilities, a calibration procedure is required. The calibration inevitably leads to inconvenience for users, especially for users with disabilities. Thus, cross-session and subjects transfer learning has been considered an important research direction to avoid such inconvenience.

Kwon et al. (2020) constructed a large MI-EEG database and proposed a subject-independent framework underlying CNN. Kant et al. (2020) proposed using CWT transforms one-dimensional EEG signals into two-dimensional time-frequency-amplitude representation enabling us to exploit available deep networks through transfer learning (Kant et al., 2020). Hu et al. (2021) proposed the Multi-Feature Fusion Method based on Wavelength Optimal Spatial Filter and Multiscale Entropy. The method can combine wavelength features with multiscale entropy. Yang L. et al. (2021) employ raw multi-channel EEG as inputs, to boost decoding accuracy by the channel-projection mixed-scale convolutional neural network (CP-MixedNet) aided by amplitude-perturbation data augmentation. Li et al. (2019) and An et al. (2020) propose a novel two-way few shot network that is able to efficiently learn how to learn representative features of unseen subject categories and how to classify them with limited MI EEG data. Yang L. et al. (2021) propose a discriminative feature learning strategy to improve the discrimination of features, which includes the central distance loss (CD-loss), the central vector shift strategy, and the central vector update process. Wei X et al. propose a multi-branch deep transfer network, the Separate-Common-Separate Network (SCSN) based on splitting the network’s feature extractors for individual subjects (Wei et al., 1999).

## Multi-Hierarchical Representation Fusion Model for Session-To-Session Motor Imagery Task

This section describes the collection process for raw EEG signals and its preserving representation primarily. Also, the proposed approach is described in detail. We propose the cascade SSAE framework fusion with the temporal and spectral features in sequence to exploit a subject-invariant representation from the adversarial source domain training. Last, the cross-sessions and subjects training are executed.

### Data Acquisition and Its Preserving Form

**FIGURE 1:**
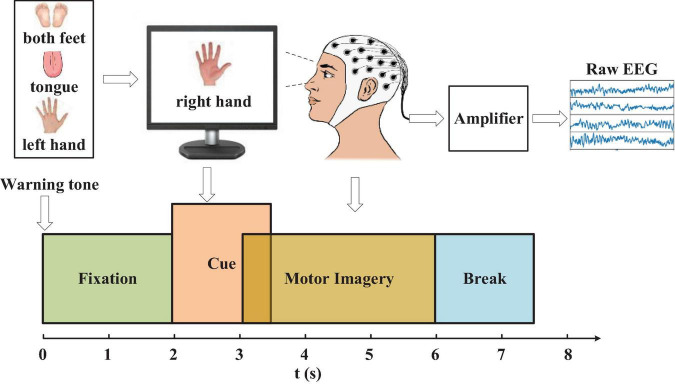
The experimental paradigm. The recording was divided into 4 blocks: (1) 2s with eyes open (looking at a fixation cross on the screen) after a warning tone, (2) A visual cue of different MI task appeared on the screen for 1.5 s (3) The subjects were asked to carry out the motor imagery task until the cue of MI finished at *t* = 6s. (4) A short break followed.

Our approach is evaluated on our constructed dataset and public dataset. We collected a dataset with the g.tec portable EEG Acquisition System (16 electrodes 10-20 system configuration). This experimental implementation involves six healthy subjects (SubA- SubF) with a mean age of 25 years being asked to wear the EEG device and sit in front of a computer screen with guidance. Four different MI tasks were conducted with a visual cue measure: left hand, right hand, both feet, and tongue. The entire experimental paradigm is illustrated in Figure 1. The cue-based BCI paradigm was composed of three sessions on different days recorded for each subject. Each session consisted of 6 runs separated by short breaks. One run was comprised of 48 trials (12 for each of the four possible classes), yielding a total of 288 trials per session. Both dates were stored at a 250-Hz sample rate. In addition to our own constructed dataset, a public BCI competition IV dataset 2a is also employed. The dataset is a 22-electrode EEG motor-imagery dataset, with nine subjects and two sessions, each with 288 4-s trials of imagined movements per subject (movements of the left hand, the right hand, the feet, and the tongue) (An et al., 2020). The training set consists of the 288 trials of the first session, and the test set consists of the 288 trials of the second session. The detailed data are summarized in Table 1. An additional 50-Hz notch filter was enabled to suppress line noise. In this paper, C3, C4, and Cz (Table 1) channels are selected. EEG measurements are infected with external and cognitive noises that impede further analysis due to unwanted effects. Moreover, crosstalk also degrades the MI EEG data patterns due to interference from neighboring electrodes. To avoid these effects, in this study the filtering technique is employed. In the step, EEG signals are band-pass filtered with 7–30 Hz to retain the (7–14 Hz) and (17–30 Hz) bands as these two bands have information related to imagine movement.

**TABLE 1:** Properties of raw materials.

Datasets	Public	Private

	D1	D2
Subjects	9	6
Sample rate	250 Hz	250 Hz
Imagery task	Left hand, right hand, both feet, tongue	Left hand, right hand, both feet, tongue
Sessions	2	3
Trials/session	288	300

### Overview of the Proposed Approach

**FIGURE 2:**
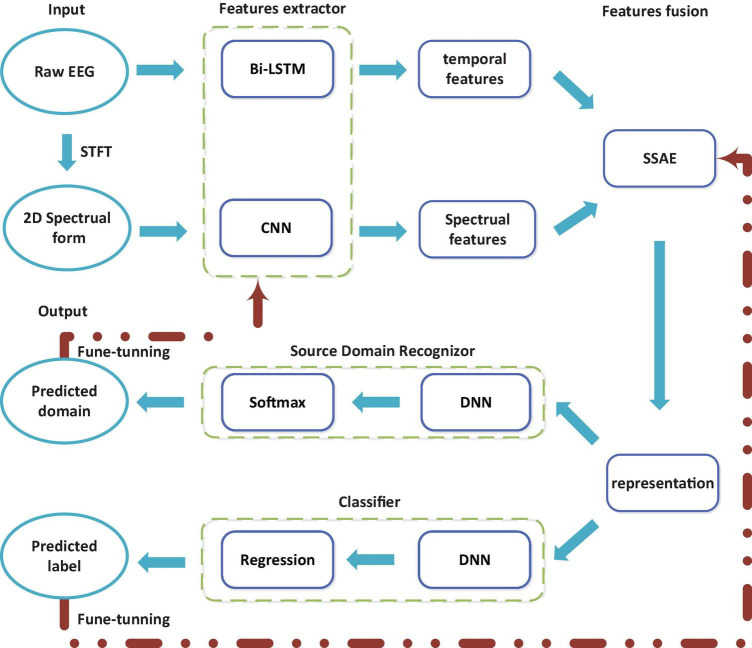
Schematic overview of the proposed multi-hierarchical discriminative deep learning (MDDL) method.

Figure 2 illustrates the steps of the proposed multi-hierarchical discriminative deep learning (MDDL) architecture. The proposed deep learning model is designed to improve generalization and robust capability by capturing the invariance representation based on MI-EEG data from different sessions. To obtain useful and informative EEG features, a parallel feature learning method combining Bi-LSTM with CNN is employed to tackle the EEG sequence and its 2D transformation by STFT. Bi-LSTM is conductive to extracting the contextual correlation of sequential form, while CNN is well benefited for the 2D time-spectral data representation.

### Multi-Hierarchical Representation Architecture

**FIGURE 3:**
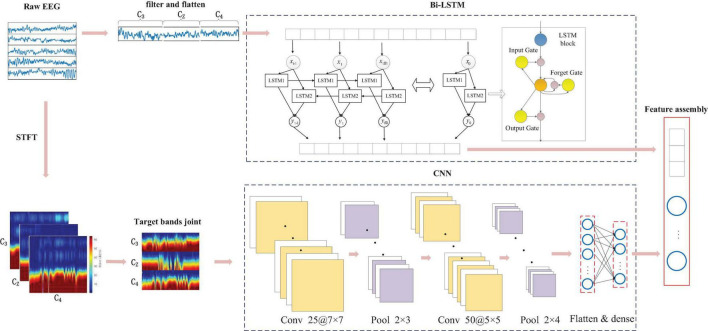
Parallel BLSTM and convolutional features fusion architecture. Bidirectional LSTM (Bi-LSTM) and convolutional neural network (CNN) were employed to learn the different hierarchical representation from raw data, then reconstruct the fusion features through stacked sparse autoencoder (SSAE).

It was demonstrated by considerable experiments that the four-class task (left hand, right hand, both feet, and tongue movement) of MI is highly related to the ERD/ERS phenomenon of the three channels (C3, C4, and Cz) (Yang et al., 2018). To view the dynamic dependency and capture multi-hierarchical high-level representation, we construct a parallel bidirectional long-term and short-term memory cyclic neural network (BLSTM) and convolutional neural network, as shown in Figure 3.

#### Bidirectional Long-Term and Short-Term Memory Cyclic Neural Network for Dynamic Contextual Feature Learning

We first propose cascade inter-channel representation and Bi-LSTM to capture the contextual correlation from either the sequential point or the dynamic interdependencies of spatial channels. Recently, an LSTM network gained popularity because of its capability to learn the long-term dependencies of sequential information (Salehinejad et al., 2018), which is definitely beneficial for temporal feature processing. In addition, they can effectively address the vanishing gradient problem in the series data (Liang et al., 2020) *via* temporal shortcut paths. A standard LSTM block consists of input, forget, and output gates and a cell activation component. Its gates can inhibit the rest of the network from modifying the contents of the memory cells for long-term timesteps.

Taking the fact that LSTM can also process data in the previous order, bi-directional LSTM was proposed to process data in both forward and backward directions with two separate hidden layers (Rui et al., 2017). Owing to these networks theoretically involving all information of input sequences during computation, and furthermore each LSTM block’s maintenance of independent parameters despite its identical input signal. Thus, the use of a Bi-LSTM network at each time step is conducive to sequence processing. As shown in Figure 3, LSTM1 only preserves the correlation of previous EEG signals, while the reversed LSTM2 can preserve the correlation of future EEG signals. Thus, the LSTM1 and LSTM2 are used to learn the forward and backward signals to capture correlational (current, previous, and future) features, especially the channel-to-channel dependency. The Bi-LSTM is applied to three electrodes signal with each 2 s long MI-task trial. We borrow learning functions defined in Ortiz-Echeverri et al. (2019) as follows:


(1) $${}_{\mathrm{Future}:\left\{ \begin{array}{c} \vec{i_{t}}=\sigma_{g}\left(\vec{W_{c}}x_{t}+\vec{U_{i}}\vec{h_{t-1}}+\vec{b_{i}}\right)\\ \vec{f_{t}}=\sigma_{g}\left(\vec{W_{f}}x_{t}+\vec{U_{f}}\vec{h_{t-1}}+\vec{b_{f}}\right)\\ \vec{o_{t}}=\sigma_{g}\left(\vec{W_{o}}x_{t}+\vec{U_{o}}\vec{h_{t-1}}+\vec{b_{o}}\right)\\ \vec{c_{t}}=\vec{f_{t}}\text{e}\vec{c_{t-1}}+\vec{i_{t}}\text{e}\sigma_{t}\left(\vec{W_{c}}x_{t}+\vec{U_{c}}\vec{h_{t-1}}+\vec{b_{c}}\right)\\ \vec{h_{t}}=\vec{o_{t}}\text{e}\sigma_{t}\left(\vec{c_{t}}\right) \end{array}\right.}$$

(2) $${}_{\mathrm{Previous}:\left\{ \begin{array}{c} \overset{\leftarrow }{i_{t}}=\sigma_{g}\left(\overset{\leftarrow }{W_{i}}x_{t}+\overset{\leftarrow }{U_{i}}\overset{\leftarrow }{h_{t-1}}+\overset{\leftarrow }{b_{i}}\right)\\ \overset{\leftarrow }{f_{t}}=\sigma_{g}\left(\overset{\leftarrow }{W_{f}}x_{t}+\overset{\leftarrow }{U_{f}}\overset{\leftarrow }{h_{t-1}}+\overset{\leftarrow }{b_{f}}\right)\\ \overset{\leftarrow }{o_{t}}=\sigma_{g}\left(\overset{\leftarrow }{W_{o}}x_{t}+\overset{\leftarrow }{U_{o}}\overset{\leftarrow }{h_{t-1}}+\overset{\leftarrow }{b_{o}}\right)\\ \overset{\leftarrow }{c_{t}}=\overset{\leftarrow }{f_{t}}\text{e}\overset{\leftarrow }{c_{t-1}}+\overset{\leftarrow }{i_{t}}\text{e}\sigma_{t}\left(\overset{\leftarrow }{W_{c}}x_{t}+\overset{\leftarrow }{U_{c}}\overset{\leftarrow }{h_{t-1}}+\overset{\leftarrow }{b_{c}}\right)\\ \overset{\leftarrow }{h_{t}}=\overset{\leftarrow }{o_{t}}e\sigma_{t}\left(\overset{\leftarrow }{c_{t}}\right) \end{array}\right.}$$

(3) $$\mathrm{Output}:{}_{y_{t}=\vec{h_{t}}\text{e}\overset{\leftarrow }{h_{t}}}$$

where *W*, *U*, and *b* refer to the weight matrices, recurrent weight matrices, and bias of different components, respectively. *i_*t*_, f_*t*_, o_*t*_, c_*t*_*, and *h*_*t*_ denote the result of the input gate, forget gate, cell candidate, output gate, and hidden state at time step *t* in sequence. σ_*g*_ and σ_*t*_ represent sigmoid and tanh activation functions. Moreover, *e* stands for the Hadamard product.

#### Convolutional Neural Network for 2D Time-Frequency Image-Form Learning

Although Bi-LSTM has the advantage of exploring the contextual (inter-sample and inter-channel) relevance in MI-EEG sequence, it is unavailable for appropriate decoding spectral (intra-frequency) representations, regarded as the most direct reflection of ERS/ERD phenomenon. To exploit discriminating features from μ and β rhythm, we mapped each MI-task EEG signal to the 2D time-frequency power form through STFT. Further, these format dates are fed into pre-designed CNN. STFT was also applied to the time series for each 2 s long MI-task trial (totally 500 signal points), with window size set to 40 and time-lapses set to 4. Considering the importance of the μ-band and β-band in the four-class MI task, in this paper, we adopt 7–14 Hz frequency bands to represent the μ-band with two resolution calculations at each frequency in STFT. Short-time Fourier transform was employed on the time sequence for each 2-s trial which is equal to 500 samples. Short-time Fourier transform was performed with window size corresponding to 50 and time lapses equal to 5. Starting from sample 1 toward sample 500, STFT is almost computed for 90 windows over 500 samples. Then we extracted beta frequency bands from the output spectrum. The frequency bands between 7 and 14 and 17–30 were considered to represent mu and beta bands. The frequency bands are slightly different than in the literature, but they resulted in a better data representation in our experiments. Taking the consideration of the MI effect from mu and beta, the size of the extracted image for the mu band was reshaped to 90 × 16 where the size of the extracted image for the beta band was 90 × 14. Accordingly, the input image formats of three spatial electrodes (C3, Cz, and C4) for μ and β (17–30 HZ) band are 90 × 90, the square matrix image matching with CNN appropriately.

In a typical CNN (Ioffe and Szegedy, 2015) process, inputs are convolved with several multidimensional kernels in the convolutional layer and subsampled to a smaller size in the pooling layer. Parameters of CNN are learned through the back-propagation algorithm, optimizing the classifier. Input data, time, frequency, and electrode location information of MI-task EEG are mapped together into a 2D image form in this paper. The vertical representation (spectral and spatial information) on the input image plays a more important role than the horizontal information in the recognition task. Thus, we introduced CNN to reinforce the function of filtering the horizontal information. The employed CNN is comprised of six layers: convolutional, pooling, and fully connected layers, as depicted in Figure 3. The entire convolutional process is stated in Table 2. The number of filters in the first and second convolutional later are empirically set to 25 and 50, respectively. The 50 feature maps obtained through the two convolution layers have a size of 19 × 16. Each convolutional block involves one batch normalization (BN) (Nair and Hinton, 2000) following a rectified linear unit (ReLU) activation (Yang et al., 2020). At the convolution layer, the input image convolved to form the *k*-th filter at a given layer and is defined as:


(4) $$a_{i,j}=f\left({\left({\text{W}}_{k}*x\right)}_{ij}+{\text{b}}_{k}\right)$$

where **W***^k^* and **b***_*k*_* represent the weight and the bias item, and *f*(*) denotes the ReLU activation function.

**TABLE 2:** The hyperparameter of the proposed convolutional neural network (CNN).

Layers	Input	Kernel	Stride	Output	Operation	Parameter number
C1	90 × 90	7 × 7	1 × 1	84 × 84 × 25	25@filters Conv2D	1250
P1	84 × 84	−	2 × 3	42 × 28 × 25	2 × 3 Max-pooling	−
C2	42 × 28 × 25	5 × 5	1 × 1	38 × 24 × 50	50@filters Conv2D	1300
P2	38 × 24 × 50	−	2 × 4	19 × 6 × 50	2 × 4 Max-pooling	−
F1	19 × 6 × 50	−	−	5700	flatten	−
F2	5700	−	−	60	Dense	342060

### Adversarial Architecture for Invariance Capturing

In this section, we propose an adversarial architecture for feature fusion to generate the multi-hierarchical representation of data. Useful information is extracted during the processes of building classifiers or other predictors.

#### Invariant High-Level Feature Construction

**FIGURE 4:**
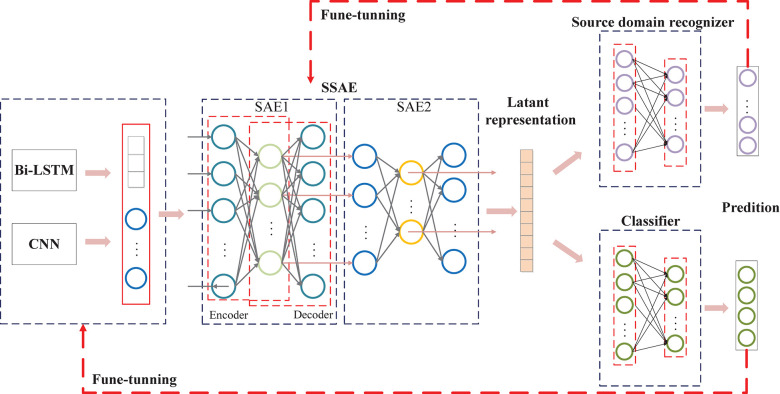
The proposed domain-adversarial network architecture for exploiting invariance. Domain discriminant model and class identification model are both trained based on the reconstructed features. The difference is domain discriminant model try to obtain the invariant features cross-session or cross-subject while class identification model try to capture the discriminative information for classification.


First, we introduce unsupervised feature learning, the SSAE (Figure 4), to further interpret EEG signals (Gogna et al., 2017). The SSAE is trained in an end-to-end learning manner to determine the more appropriate model for MI–EEG signals. Meanwhile, the output of the encoder can also be used as integrated features for EEG decoding. The data transformation procedure of SSAE can be defined as:

(5) $$\left\{ \begin{array}{c} \text{H}=\sigma \left({\text{W}}_{en}\text{X}+{\text{b}}_{en}\right)\\ {\text{X}}^{\prime }=\sigma \left({\text{W}}_{de}h+{\text{b}}_{de}\right) \end{array}\right.$$

where *W*_*en*_, *W*_*de*_, *b*_*en*_, and *b*_*de*_ indicate the weights and biases in the encoder and the decoder. *h* and *X′* denote the hidden layer and output (reconstruction) layer vector, respectively. In this study, the input is the combination of two hierarchical representations. The mean squared error (MSE) is used as the cost function, and the backpropagation is used to optimize the weights and biases. Multi-layer representation in deep learning can yield more general and beneficial features (Ajakan et al., 2016). We introduce a sample architecture of SSAE with two SAEs to capture invariance for later adversarial networks.

#### Domain-Adversarial Networks

Domain-adversarial networks inspired by relevant references (Zhang et al., 2018; Kwon et al., 2020) are employed to enable a model with splendid generalizing capability from one domain to another. Simultaneously, we ensure the internal representation of the network discriminative information referring to the origin of the input (source or target) while preserving a low risk on the source samples. A classifier is constructed for the source domain, being pre-trained with the different source domain data and learning to discriminate them. The goal is balancing between source and task domain discriminator through domain-adversarial training. Note that it is deemed that the invariance capturing from different source domains has been achieved when source domain recognizer confused (maximum domain cost) accompanied with the task classifier has a satisfactory discriminative performance (minimum classifier cost).

The proposed domain-adversarial network is illustrated in Figure 4. The *G*_*m*_ learns a function: **X**→**F***^D^* maps EEG samples into a new *D*-dimensional feature from multi-hierarchical Bi-LSTM and CNN. Then *G*_*S*_ learns a function: **F***^D^*→**R***^D^* constructs latent representation from multi-hierarchical features. They are defined in a matrix-vector form as follows:


(6) $$\text{F}=G_{m}\left(X;{\text{W}}_{m},{\text{b}}_{m}\right)$$

(7) $$\text{R}=G_{s}\left(F;{\text{W}}_{s},{\text{b}}_{s}\right)$$

The prediction of classifier maps a function *G*_*y*_:**R***^D^*→[0,1,2,3], which is parameterized by:


(8) $$G_{y}\left(G_{s}\left(G_{m}\left(X\right)\right);W_{y},b_{y}\right)=\mathrm{softmax}\left(W_{y}G_{s}\left(G_{m}\left(X\right)\right)+b_{y}\right)$$

with:


(9) $$\mathrm{softmax}\left(\alpha \right)={\left[\frac{\exp\left(a_{i}\right)}{\sum_{j=1}^{\left|\text{a}\right|}\exp\left(a_{j}\right)}\right]}_{i=1}^{\left|\alpha \right|}$$

Given the labeled source {*x*_*i*_, *y*_*i*_}, the used classification loss is the negative log-probability of the correct label:


(10) $$L_{y}\left(G_{s}\left(G_{m}\left(x_{i}\right)\right),y_{i}\right)=\log\frac{1}{G_{s}{\left(G_{m}\left(x_{i}\right)\right)}_{y_{i}}}$$

The neural network is trained for the *i*-th sample, which then leads to the following optimization problem:


(11) $$\min_{W_{m},W_{y},b_{m},b_{y}}\left[\frac{1}{n}\sum_{i=1}^{n}L_{y}^{i}\left(W_{m},b_{m}\right)+\lambda \theta \left({\text{W}}_{s},{\text{b}}_{s}\right)\right]$$

where θ(**W***_*s*_*, **b***_*s*_*) presents an optional regularizer to be described below.

For a domain classification, *G*_*d*_, learning a logistic regressor: **R***^D^*→[0,1,…,n] that models the probability for a given input from the source domain (session or subject). Thus:


(12) $$G_{y}\left(G_{s}\left(G_{m}\left(X\right)\right);{\text{W}}_{d},{\text{b}}_{d}\right)=\mathrm{sigm}\left(W_{d}G_{s}\left(G_{m}\left(X\right)\right)+{\text{b}}_{d}\right)$$

Then, the adversarial source domain loss is defined by:


(13) $$L_{d}\left(G_{d}\left(r_{i}\right),d_{i}\right)=d_{i}\log\frac{1}{G_{d}\left(r_{i}\right)}+\left(1-d_{i}\right)\log\frac{1}{1-G_{d}\left(r_{i}\right)}$$

where *r*_*i*_ and *d*_*i*_ denote the mapping representation for the *i*-th EEG samples. In view of a domain adaptation for the entire training, we added the regularizer term to the global cost as:


(14) $$\theta \left({\text{W}}_{s},{\text{b}}_{s}\right)=-\frac{1}{n}\sum_{i=1}^{n}L_{d}^{i}\left({\text{W}}_{s},{\text{b}}_{s}\right)-\frac{1}{n^{\prime }}\sum_{i=n+1}^{N}L_{d}^{i}\left(W_{s},b_{s}\right)$$

The optimization objective (11) is rewritten to:


(15) $$\begin{array}{c} E\left({\text{W}}_{m},{\text{W}}_{s},{\text{W}}_{y},{\text{W}}_{d},{\text{b}}_{m},{\text{b}}_{s},{\text{b}}_{y},{\text{b}}_{d}\right)\\ =\frac{1}{n}\sum_{i=1}^{n}L_{y}^{i}\left({\text{W}}_{m},{\text{b}}_{m}\right)-\lambda \\ \left(\frac{1}{n}\sum_{i=1}^{n}L_{d}^{i}\left({\text{W}}_{s},{\text{b}}_{s}\right)+\frac{1}{n^{\prime }}\sum_{i=n+1}^{N}L_{d}^{i}\left({\text{W}}_{s},{\text{b}}_{s}\right)\right) \end{array}$$

The optimization problem involves a minimization with respect to classification parameters, as well as a maximization in accordance with the source domain discriminating ones:


(16) $$\begin{array}{c} \left(\overset{∼}{\underset{m}{\text{W}}},\overset{∼}{\underset{y}{\text{W}}},\overset{∼}{\underset{m}{b}},\overset{∼}{\underset{y}{b}}\right)\\ =\underset{W_{m},W_{y},b_{m},b_{y}}{\arg\min}E\left({\text{W}}_{m},W_{s}^{∼},{\text{W}}_{y},\overset{∼}{\underset{d}{W}},{\text{b}}_{m},\overset{∼}{\underset{s}{b}},{\text{b}}_{y},\overset{∼}{\underset{d}{b}}\right) \end{array}$$

(17) $$\begin{array}{c} \left(\overset{∼}{\underset{s}{W}},\overset{∼}{\underset{d}{W}},\overset{∼}{\underset{s}{b}},\overset{∼}{\underset{d}{b}}\right)\\ =\underset{W_{m},W_{y},b_{m},b_{y}}{\arg\max}E\left(\overset{∼}{\underset{m}{W}},{\text{W}}_{s},\overset{∼}{\underset{y}{W}},{\text{W}}_{d},\overset{∼}{\underset{m}{b}},{\text{b}}_{s},\overset{∼}{\underset{y}{b}},b_{d}\right) \end{array}$$

where ∼ represents the optimal parameters. Max-min optimization explores the latent representation in the dynamic balance situation where the task classifier can work effectively when the domain recognizer makes confusion. It implies that the proposed framework gains invariance from different source domains.

## Experiments

This study mainly adopted inter-session and inter-subject validation types to compare with the cross-validation baseline. Cross-sessions validation used one session data as a testing set and all the rest as a training set. The inter-session training methodology for MI-based BCI is considered more challenging about session information transfer, playing an extremely critical role in developing calibration-free BCI with generalization and robustness. A similar validation strategy was also used in the inter-subject validation, leave-one-subject-out executions. Aiming at the analysis of the calibration situation, we additionally introduce semi-transfer validation strategies.

### Cross-Validation of Multi-Hierarchical Representation Fusion

First, we conducted fourfold cross-validation to evaluate MHRF without a domain-adversarial process (only a classifier for perdition about task label) as a baseline comparison. The proposed model was implemented in Python. Table 3 summarizes the accuracy for all sessions and subjects. As shown in Table 3, all subjects have a good performance in the MI task except S6 in D2. Among the three sessions in D2, outperforming session data amid them (bold marks) would be utilized as the test set in cross-session transfer validation and the remainders as the training sets.

**TABLE 3:** Average accuracy of multi-hierarchical representation fusion (MHRF) in cross-validation.

Subjects	D1		D2		
	Session 1	Session 2	Session 1	Session 2	Session 3
S1	85.6 ± 7.6	82.4 ± 3.0	81.6 ± 4.5	83.6 ± 4.5	**84.6 ± 4.5**
S2	76.6 ± 8.1	79.6 ± 11.5	70.0 ± 7.3	**80.3 ± 6.3**	77.3 ± 3.2
S3	81.9 ± 5.7	86.4 ± 8.3	73.4 ± 6.1	81.5 ± 4.3	**83.3 ± 10.2**
S4	80.3 ± 8.9	81.2 ± 4.5	79.0 ± 7.3	**80.3 ± 4.3**	76.3 ± 7.2
S5	74.2 ± 7.5	80.6 ± 3.5	**83.4 ± 8.1**	76.5 ± 4.3	81.3 ± 10.2
S6	78.6 ± 10.2	75.6 ± 6.5	53.0 ± 7.3	43.3 ± 7.3	47.3 ± 5.2
S7	81.6 ± 8.3	77.6 ± 8.2			
S8	81.6 ± 3.9	75.6 ± 9.2			
S9	83.6 ± 5.5	84.6 ± 5.7			
AVG.	80.3 ± 7.14	80.6 ± 6.7	73.4 ± 6.8	74.3 ± 5.2	75.0 ± 6.8

*Bold values indicates best result of the subject.*

### Session-to-Session Validation

#### Validation of Transfer Capacity in D1

**FIGURE 5:**
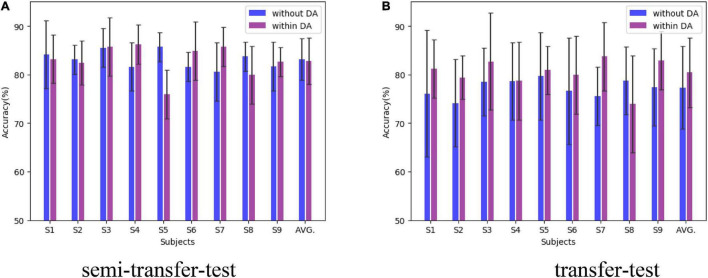
Comparison of transfer capacity among subjects in D2. The black line upper and lower indicate the maximum and minimum deviation value.

With the consideration of two sessions in D1 only, lacking domains, and the purpose of exploring the transfer capacity, we randomly choose half session 1 data as the testing set and the remaining one of session 1 and session 2 data as the training set. Only session 2 is also utilized as the training set. Those were successively expressed as semi-transfer-test and transfer-test. Such training strategy was applied to implementation without and within domain-adversarial (marked as DA) for comparison. Two sessions’ data were equally divided into four domain parts for domain-adversarial training. Figure 5A illustrates the semi-transfer-test results, where the MHRF underlying DA training maintained outstanding performance compared with DA training, both in the semi-transfer-test and transfer-test. Moreover, we found negative transfer evidence in S5 and S8 (obviously degraded performance for the introduction of the transfer method).

#### Different Methods on D2

**FIGURE 6:**
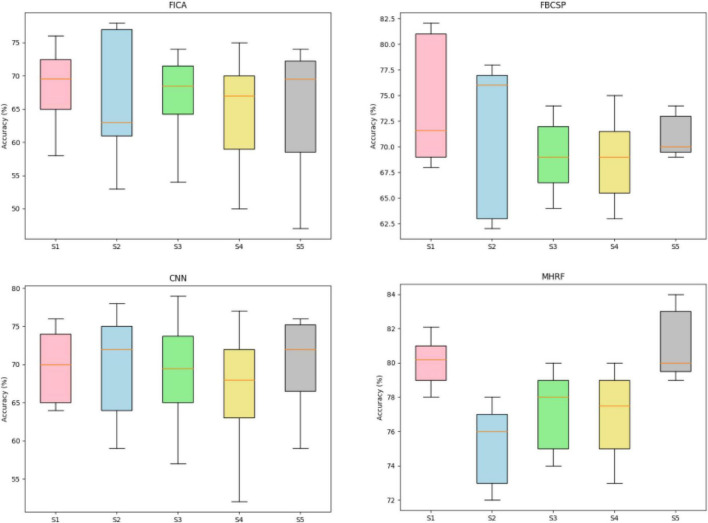
The inter-session transfer accuracies applied to five subjects (S1-S5) with four algorithms (FICA, FBCSP, CNN, and MHRF). The brown line Brown lines indicate the median values and the upper and lower edges of the box indicate the quartiles.

Figure 6 depicts the accuracy boxplots of session-to-session transfer, consisting of four accuracy boxplots achieved by different methods through inter-session transfer training strategy with the outperforming session data as the testing part. FBCSP and FICA indicate the machine learning methods for a triple channel EEG signal proposed in Zou et al. (2019). Convolutional neural network indicates only using the convolution processing for a 2D time-frequency target MI-EEG transformed by STFT. As shown in Figure 6, the MHRF outperformed average accuracy over the other machine learning algorithms. Either the FBCSP or CNN framework provided a certain comparable performance in discrimination among different subjects but was incapable of obtaining enough valuable information for inter-session transfer.

**FIGURE 7:**
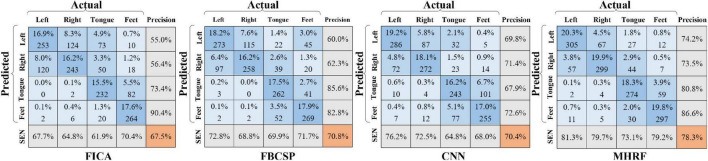
Confusion matrices for the four methods (FICA, FBCSP, CNN, and MHRF).

Figure 7 shows the confusion matrix for the four-category classification, where four similar methods were compared under the inter-session transfer training strategy. Each cell depicts the prediction accuracy (upper) and the trial numbers (lower) of the target predicted as a certain class. The dark blue diagonal corresponds to correctly predicted trials of the four classes. The orange value is the overall accuracy. In sequence, the bottom row and rightmost column represent sensitivity and precision evaluation indicators. The confusion matrices in Figure 7 show classification results for four tasks. The MHRF achieved an 8% higher accuracy than the second alternative, revealing its strong competitiveness in inter-session transfer learning. Note that relatively high sensitivity and precision in tongue and feet MI task on FICA and FBCSP indicate the advantages obtained by FICA and FBCSP in a single-channel analysis due to the discriminating information of tongue and feet, mainly contained in the Cz channel. Also, a high score of left- and right-hand MI on CNN and MHRF (including CNN) indicates the superiority of the CNN and MHRF in inter-channel processing (C3 and C4).

#### Subject-To-Subject Validation

**FIGURE 8:**
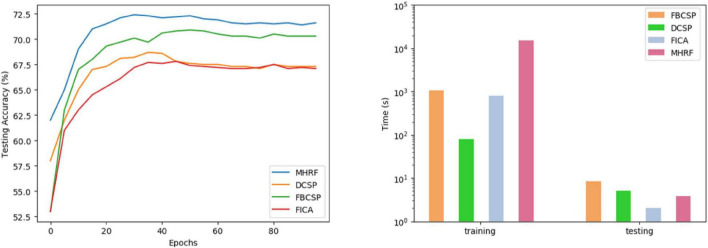
Training and its corresponding time consumption plot for leave-one-out executions of the four algorithms on D1.

In this experiment, we evaluated the classification accuracy on an inter-subject validation basis. Specifically, one subject from the dataset in MI-task is used as the test subject, and the remaining subjects are used as training subjects. Each subject is assumed to constitute his own domain to gain multiple source domains. In total, one subject offered 288 samples (near 72 samples/class), and the training set in D1 consisted of 2304 samples from 8 subjects. The test set consisted of 288 samples from the test subject. The plot on the left in Figure 8 presents the testing accuracy of MHRF over training epochs. The graphs show that MHRF has peaked at 30 epochs with its alternatives at 50, 38, and 42, respectively, indicating the fast-converging capacity of the proposed framework. In addition, the low overall accuracy in inter-subject transfer learning shows the challenges in individual variability. The plot on the right in Figure 8 compares the computational time in inter-subject training and testing. The computational complexity of the MHRF is the highest among compared models due to its parallel feature extraction structure and adversarial invariance capturing pattern. However, training is a one-off operation. For practical considerations, the inference time during testing is the most crucial factor. The MHRF runs less than 1 s, similar to other compared methods.

**FIGURE 9:**
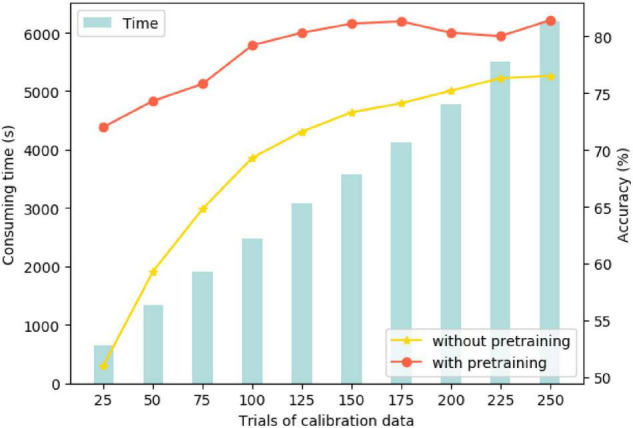
Training accuracy and calibration time in both modes.

Two models were compared to explore the calibration process: One was the trained inter-subject model as the initial pre-trained model to introduce the target data (testing subject) for calibration, called “pretraining.” The other comparative one was the direct training with the target data labeled without pretraining. Figure 9 illustrates the recognition accuracy and calibration time. On the ground of line and bar plot, the calibration accuracy of the model with pretraining increased to about 80% after 100 epochs (near one-third) of target data learning. In comparison, the model without pretraining only reached 76% after the entire epoch completion. This suggests that the pretraining model can serve the target data calibration more efficiently, obtained using non-homologous data under the same task. Moreover, in such a way, we can use partial and a small amount of available data. The computational time for the calibration process is linearly increased in accordance with the amount of target data trials.

#### Visualization Analysis of Deep Feature Fusion

**FIGURE 10:**
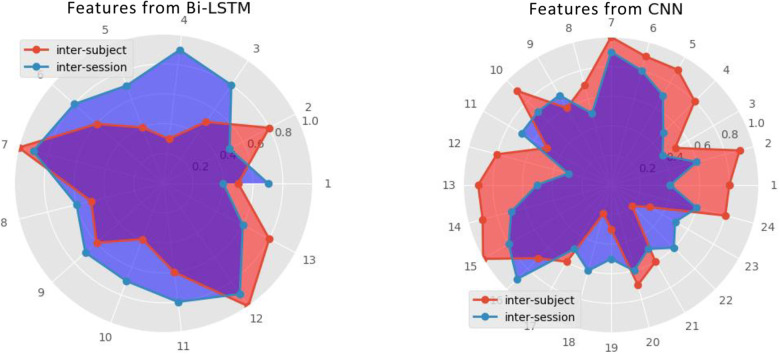
The comparison of feature fusion in radar plot.

The radar plot (Figure 10) was used to visualize the influences of features from different frameworks (inter-session and inter-subject) on the final recognition accuracy, analyzing the fusion features. The radius represents the influential weights of features after normalization. As shown in Figure 9, the number of valid features fusion through SSAE reconstructing from Bi-LSTM and CNN is 13 and 24, respectively. It reveals that MI recognition is more sensitive to 2D representation from CNN. Another interesting discovery is the larger coverage area of inter-session on features from B-LSTM. The opposite appears on another radar plot about features from CNN. They indicate more dependency of the invariance capturing on inter-session transfer learning on features extracting from Bi-LSTM framework, while simultaneously more reliance of inter-subject one on features transformed from CNN.

## Discussion

The EEG data we employed on cross-session and subject decoding in the experiments include 6 participants (private data) and 9 subjects (public data) with 288 and 300 samples from each subject, respectively (Table 1). The performance of classification accuracies on them were reported in Table 3, that guaranteeing the data can provide the task identification characteristics as a basis in advance.

We have introduced a framework for transfer learning, on both cross-sessions and subjects, that works through capturing invariant features and achieving better performance than other state-of-the-art methods for classification. Furthermore, the proposed MHRF framework enables us to make a direct and intuitive assessment on the performance in terms of classification accuracies. MHRF is a deep learning network which was inspired by both multi-hierarchical feature fusions. Specifically, unlike conventional feature fusions, it creates representation by incorporating a domain adversarial adaptive part. As shown in Figures 5–7, MDDL maintains robust on cross-session transfer. First, MHRF outperforms both in the semi-transfer-test and transfer-test, shown in Figure 5. Especially with domain-adversarial process, the MHRF may be more distinguishable along with achieving a 3% higher average accuracy. Furthermore, also in consideration of cross-session data, MHRF is outperforming in accuracy (Figure 6) and further confusion matrix (Figure 7) compared with FBCSP, FICA, and conventional CNN all around. There was an 8% over higher percentage than the second alternative method in accuracy.

To compare our MHRF algorithm with other methods more rigorously in transitivity, we further analyzed its effectiveness by executing experiments on cross-subjects’ data. Figure 8 shows that the averaged convergence accuracy across all subjects is 71.7%, 70.0%, 66.7%, and 66.6%, respectively. Meanwhile Figure 8 shows the comparison of the computational time for training and testing cross-subjects. MHRF takes more time in training but performs effectively in testing. Two calibration pattern comparisons are shown in Figure 9 and we found that the pretraining model could serve the target data calibration more efficiently, which guides us to utilize partial and a small amount of available data for the construction of a highly efficient subject-to-subject decoding system. Finally, we have explored the correlation between multi-hierarchical features and cross-data pattern.

## Conclusion

This paper proposed a novel MHRF method that attempts to learn invariant representations from non-stationary EEG data across different subjects and sessions. Bi-LSTM and CNN were employed to learn both temporal dynamic-correlation and spatial-spectral information. We constructed an unsupervised SSAE trained in an adversarial manner to transform the extracted features into a domain-invariant subspace representation, ensuring the generalization of recognition among sessions and subjects. Some novel training strategies are also introduced, such as semi-transfer-test and transfer-test. The experiments on both public and our own constructed datasets show the feasibility and effectiveness of the proposed MHRF model on inter-session learning. Further, the proposed model is proved to have advantages and robustness in inter-subject calibration with partial and a small amount of available target data. Our further works will include exploring the domain-discrepancy reducing strategy.

## Data Availability Statement

The original contributions presented in the study are included in the article/supplementary material, further inquiries can be directed to the corresponding author.

## Ethics Statement

The studies involving human participants were reviewed and approved by Graz University of Technology, Austria. The patients/participants provided their written informed consent to participate in this study.

## Author Contributions

TS and JY conceived of the presented idea. JY developed the theory and performed the computations. JY and LL designed and conducted the experiments. JY, TS, and HY analyzed the data and wrote the manuscript. All authors contributed to the article and approved the submitted version.

## Conflict of Interest

The authors declare that the research was conducted in the absence of any commercial or financial relationships that could be construed as a potential conflict of interest.

## Publisher’s Note

All claims expressed in this article are solely those of the authors and do not necessarily represent those of their affiliated organizations, or those of the publisher, the editors and the reviewers. Any product that may be evaluated in this article, or claim that may be made by its manufacturer, is not guaranteed or endorsed by the publisher.
